# Activated cytotoxic T cells within zoonotic cutaneous leishmaniasis lesions

**DOI:** 10.1002/iid3.240

**Published:** 2019-04-17

**Authors:** Thouraya Boussoffara, Mohamed Samir Boubaker, Melika Ben Ahmed, Mourad Mokni, Salma Feriani, Afif Ben Salah, Hechmi Louzir

**Affiliations:** ^1^ Laboratory of Transmission Control and Immunobiology of Infections, Pasteur Institute of Tunis Tunis Tunisia; ^2^ Université de Tunis El Manar Tunis Tunisia; ^3^ Laboratory of Human and Experimental Pathology Pasteur Institute of Tunis Tunis Tunisia; ^4^ Faculté de Médecine de Tunis Tunis Tunisia; ^5^ Department of Dermatology Hospital La Rabta Tunis Tunisia; ^6^ Department of Family and Community Medicine College of Medecine and Medical Sciences, Arabian Gulf University (AGU) Manama Bahrain

**Keywords:** cytotoxic cells, granulysin, granzyme B, IFN‐γ, Leishmaniasis lesion, Leishmania major

## Abstract

**Introduction:**

Zoonotic cutaneous leishmaniasis (ZCL), due to infection by *Leishmania (L). major*, is characterized by polymorphic clinical manifestations which could be attributed to the host's immune response. In this study we investigated the involvement of cytotoxic cells on the outcome of the disease.

**Methods:**

Expression of granzyme B (GrB), granulysine (Grly), and interferon (IFN)‐γ was evaluated within ZCL lesion specimens using the technique of real‐time quantitative polymerase chain reaction (RT‐qPCR). Immunohistochemical staining was performed using anti‐CD3, CD4, CD8, CD56, GrB, and IFN‐γ antibodies to identify the phenotype of GrB and IFN‐γ‐producing cells.

**Results:**

GrB and Grly mRNA was detected within 75% and 80% of ZCL lesions, respectively. Statistical analysis demonstrated a significant correlation between levels of GrB and Grly. Interestingly, expression of these molecules correlates negatively with the lesion's age. The highest levels were measured in early lesions (E‐ZCL) (lesion age ≤1 month) comparing to late lesions (L‐ZCL) (lesion age >1 month). Otherwise, IFN‐γ mRNA was detected only within 56% and a positive correlation was found between levels of this cytokine and those of GrB. Immunohistochemical analysis showed that GrB is produced essentially by CD8^+^T cells whereas IFN‐γ is produced by both CD4^+^ and CD8^+^T cells.

**Conclusion:**

Together our results demonstrate the presence of cytotoxic cells producing GrB and Grly within leishmaniasis cutaneous lesions.

## 1 INTRODUCTION

Zoonotic cutaneous leishmaniasis (ZCL) represents an important health problem in Tunisia, with a large spectrum of clinical manifestations.^1^ The disease is caused predominantly by *L. major* MON‐25^2^ and is transmitted by the bite of a sand fly vector belonging to the *Phlebotomus* gender and spreading at the center and the south of the country. Psammomys (P.) obesus and Meriones (M.) shawi represent the most important host reservoirs of these parasites in Tunisia.^3^

There is considerable evidence that cellular immune responses within the cutaneous lesions are of primary importance in the outcome of infection. Several studies have focused on evaluating the intralesional cytokine and chemokine gene expression in the various clinical forms of leishmaniasis.^4^, ^5^, ^6^, ^7^ In American cutaneous leishmaniasis (ACL) lesions due to *L. braziliensis* or *L. mexicana*, an effector function was suggested by the demonstration of cytokine and/or cytotoxic mediators produced by CD4^+^ or CD8^+^ cells.^8^, ^9^, ^10^, ^11^, ^12^, ^13^, ^14^ Despite the active involvement of CD8^+^T cells in the immune response to cutaneous infections in humans, the role of these cells remains controversial.^15^ An enrichment of *Leishmania*‐reactive CD8^+^T cells in older lesions suggests that they play an important role in the healing process.^16^, ^17^, ^18^ However, further data point to their implication in the development of active lesions of mucocutaneous leishmaniasis (ML)^19^, ^20^ and lesion progression in human CL due to *L.braziliensis* 10, 12, 14, 21 emphasizing on the contribution of cytotoxicity to the pathogenesis of this disease.^22^ The role of such cells during *L. major* infection in humans is not yet elucidated. Large numbers of CD8^+^ T cells have been observed not only during the acute phase of *L. major* lesions but also during the healing process.^23^ The hallmark of these cells was the production of IFN‐γ and TNF‐α as well as cytotoxic capacity.^23^

Cytotoxic T lymphocytes (CTL) can exert cytotoxicity through various mechanisms: exocytosis of lytic granula containing perforin, granzyme A/B, and/or granulysin; interaction between FasL and Fas expressed on targets cells; production of TNF or TRIAL.^24^ It has been demonstrated that *L. major* induces Th1 and CD8^+^ T cells in human patients and both responses are associated with disease resolution.^25^ A major correlate of protection appears to be the high amounts of IFN‐γ produced by CD8^+^ T cells after restimulation.^25^ In vitro studies have also demonstrated that *Leishmania*‐specific CTLs are generated upon co‐culturing human naïve T cells with antigens from *L. major* parasites.^26^ Moreover, in a previous study, we have found an increased GrB activity in patients with active infection.^27^ Indeed, in vitro cytotoxicity by peripheral blood lymphocytes on *L. major*‐infected macrophages appeared to be mediated by GrB, suggesting that CTL activity may be involved in controlling parasite growth. Likewise, CD8^+^ T cells from subjects with CL due to *L. braziliensis* exhibit a great cytotoxicity (with high production of GrB) compared to subject with subclinical infection suggesting their involvement in pathology through the killing of cells infected with *Leishmania* parasites or the expression of *L. braziliensis* antigen.^11^

We aimed in this study to explore the presence of cytotoxic cells and their activation status within human cutaneous leishmaniasis lesions due to the *L. major* infection.

## 2 MATERIALS AND METHODS

### 2.1 Lesion specimens

A total of sixteen lesion's biopsies were from patients with active ZCL (age range 7‐11 years, mean age 9.6 years) with a sex ratio equals to 0.57 (4M/7F). Three patients with multiple lesions had two or three biopsies done. Biopsies were addressed to the laboratory of Human and Experimental Pathology at the Pasteur Institute of Tunis for diagnosis of cutaneous leishmaniasis (CL). Clinical criteria including size of lesions and their age corresponding to the time between the beginning of the symptoms and the diagnosis of ZCL, were documented (Table 1). Lesions were localized in the upper or lower limbs of patients. Confirmed diagnosis of CL was based on visualization of amastigotes in direct smear of the lesions. As negative controls, skin biopsies were obtained from eight donors suffering of other disease (age range 27‐59 years, mean age 39.4 years) with sex ratio equal to 0.66. Biopsies were done after local anesthesia at the border of the lesion using a 3 mm diameter skin punch. Lesions specimens were divided into two parts: one was immediately snap‐frozen in liquid nitrogen, and the other was fixed in 10% neutral formol saline for paraffin embedding.

**Table 1:** Characteristics of lesions included in the study

	Sex	Age	Size (mm^2^)	Clinical description	Infiltrate density^a^	Plasmocytes density^a^	PNN density^a^	Granuloma score^b^	Epithelial hyperplasia^c^	Nbr of parasite^d^
ZCL1	F	2 m	314	UC	(1)	(0)	(2)	(0)	(1)	Und
ZCL2		2 m	78.5	UC	(1)	(0)	(2)	(0)	(1)	264
ZCL3		2 m	314	UC	(1)	(0)	(2)	(0)	(1)	1169
ZCL4	F	1 m	1962.5	UC	(3)‐(4)	(1)	(4)	(0)	(2)	Und
ZCL5		1 m	1962.5	UC	(4)	(2)	(4)	(1)	(2)	Und
ZCL6	F	3 m	176.6	UC	(1)‐(2)	(3)	(2)	(0)	(1)	2
ZCL7		3 m	1256	UC	(1)‐(2)	(3)	(2)	(0)	(1)	5
ZCL8		3 m	176.6	UC	(1)‐(2)	(3)	(2)	(0)	(1)	274
ZCL9	M	3 m	1256	UC	(1)	(1)	(0)	(0)	(1)	17
ZCL10	M	1 m	314	UC	(2)	(2)	(1)	(0)	(2)	Und
ZCL11	F	1 y	415.2	UC	(1)	(3)	(0)	(0)	(0)	189
ZCL12	M	2 m	314	UC	(2)	(2)	(2)	(0)	(1)	83
ZCL13	F	10 d	78.5	UC	(3)	(1)	(1)	(0)	(1)	709
ZCL14	F	3 m	415.2	UC	(2)	(1)	(3)	(0)	(2)	241
ZCL15	M	‐	‐	Active	(1)	(2)	(1)	(0)	(1)	21
ZCL16	F	3 m	176.6	UC	(1)	(2)	(2)	(0)	(1)	33

^a^ Density score of the Infiltrate; plasmocytes or polynuclear neutrophils (PNN): (0) absent, (1) slight, (2) moderate, (3) intense, (4) very intense.

^b^ Granuloma score: (0) absent; (1) desorganized.

^c^ Epithelial hyperplasia: (0) absent; (1) weak; (2) moderate.

^d^ Number of parasite, evaluated by RT‐qPCR, expressed as number of parasite per 10^5^ human cells.

UC, ulcerated and crusted lesion; d, day; m, month; y, year; Und, Undetected.

### 2.2 Granzyme B, Granulysin, and IFN‐γ gene expression

Total RNA was extracted from frozen biopsies after homogenization in 1 mL of TRIzol reagent (Gibco. BRL. Life Technologies, INC), with an Ultra‐Turax homogenizer (IKA. Works, Wilmington, NC). RNA was precipitated by isopropanol, washed with 70% ethanol, and solubilized in water according to the manufacturer's instructions. Any possible genomic DNA contamination was eliminated by treatment with deoxyribonuclease (Dnase I, Boehring Mannheim, Mannheim, Germany). Approximately 1 μg of total RNA was reverse transcribed in reaction mixture containing 1X buffer (Gibco BRL), 1.7UI/μL of RNasin (Promega), 250 μM of each dNTP, 50 μg/mL of random examers (Promega), and 20 UI/μL of M‐MLV RT (Murine‐Moloney Leukemia Virus Reverse Transcriptase, Gibco BRL) for 1 hr at 42°C. The cDNA was heated at 95°C for 10 min to inactivate the reverse transcriptase.

GrB and Grly mRNA quantification was performed by RT‐qPCR using specific primers, fluorogenic probe and “Universal PCR Master Mix” containing 10 mM Tris (pH 8.3), 50 mM KCl, 10 mM EDTA, 5 mM MgCl_2_, 100 μM dATP, dCTP, dTTP, 0.3 μM of each primers, 0.1 μM fluoregenic probe, and 1.25 U of AmpliTaq Gold (PE Applied Biosystems). The upstream and downstream primer sequences of GrB were 5′‐GCGGTGGCTTCCTGATACAA‐3′ and 5′‐GGCTCCTGTTCTTTGATATTGTGG‐3′, respectively. A GrB specific fluorogenic probe 5′ FAM‐CGACTTCGTGCTGACAGCTGGCTCAC‐TAMRA 3′ was synthesized by PE Applied Biosystems (Foster City, CA). For Grly, the upstream and downstream primers sequence were 5′‐CCTGCCTCTTGCAGCCAT‐3′ and 5′‐GGGCTCTTGCCAGGTCGTA‐3′, respectively. The Grly specific fluorogenic probe was 5′FAM‐TCTGGTCTTCTCTCGTCTGACCCTGA‐TAMRA3′. For IFN‐γ mRNA quantification was performed using qPCR according to the TaqMan procedure using the “Universal PCR Master Mix” and “Pre‐developed assay reagent Kit” (PE Biosystems, Foster City, CA).

Following an activation of the AmpliTaq Gold for 10 min at 95°C, 40 cycles of denaturation at 95°C for 15 sec and annealing and extension at 62°C for 1 min were carried out using the ABI PRISM 7700 sequence detection system (with version 1.6 software, PE Applied Biosystems). Real‐time fluorescence measurements were performed, and a threshold cycle (C_T_) value for each sample was calculated by determining the point at which the fluorescence emission reaches the log phase of product accumulation.

### 2.3 Leishmania DNA quantification

To evaluate the parasite load in the ZCL lesions, we quantify the KMP‐11 (Kinetoplastid Membrane Protein) gene encoding protein which is abundantly expressed in promastigotes (1‐2 10^6^/cell).^28^ DNA in the interphase and phenol phase from the initial homogenate of lesions specimens and TRIzol reagent was precipitated with ethanol, washed twice in a solution containing 0.1M citrate in 10% ethanol, and DNA pellet was dissolved in 8 mM NaOH according to the manufacturer's instructions. DNA quantity was estimated by measure of A_260_ nm. DNA content was evaluated using A_260_ value for double‐stranded DNA. One unit equals 50 μg of double‐stranded DNA/mL.

The upstream and downstream primer sequences of KMP‐11 were 5′‐CGCCAAGTTCTTTGCGGACAA‐3′ and 5′‐CATGATCAGGGAGCACACA‐3′, respectively. A fluorogenic probe 5′ FAM ‐CGCCCGAGATGAAGGAGCACTACG‐TAMRA3′ with a sequence located between the PCR primers was synthesized by PE Applied Biosystems (Fostr City, Calif). The PCR reaction was performed using the Taq‐Man PCR kit (PE Applied biosystems). Briefly, 50 ng of DNA from skin biopsies was added to the PCR mixture containing 10 mM Tris (pH 8.3), 50 mM KCl, 10 mM EDTA, 5 mM MgCl_2_, 100 μM dATP, dCTP, dTTP, 0.3 μM of each primers, 0.1 μM fluoregenic probe, and 1.25 U of AmpliTaq (PE Applied Biosystems). PCR reactions were performed using the ABI PRISM 7700 sequence detection system as previously described. Real‐time fluorescence measurements were taken, and a threshold cycle (C_T_) value for each sample was calculated. Standard curve were prepared using 10 fold dilutions of total purified DNA extracted from *L. major* promastigotes (4 × 10^9^). C_T_ values from skin samples were plotted on the standard curve and quantity of DNA was calculated. For calculation of parasite number in analyzed samples, we assumed that the amount of DNA per *Leishmania* parasite equals 0.1 pg.^29^ For calculation of cell number in analyzed samples, we assumed that the amount of DNA per human cell equals 6 pg.^30^ Results were expressed in number of parasites per 10^5^ human cells.

### 2.4 Immunohistochemical study

Single immunoenzyme staining was performed using the labelled streptavidin biotin visualisation system (LSAB) with LSAB‐HRP kit (DakoCytomation, Carpinteria, CA) according to the manufacturer's instructions. Briefly, sections embedded in paraffin were deparaffinized and rehydrated. Immunohistochemistry reactions were preceded by blocking peroxidase activity with 3% hydrogen peroxide for 5 min. The Slides were incubated for 60 min with anti‐CD3 (DakoCytomation, Denmark), anti‐CD8 (DakoCytomation), anti‐CD4 (DakoCytomation) and anti‐IFN‐γ (Abcam, UK) or overnight with anti‐GrB (clone GrB7, Dako) at the appropriate dilution. Sections were washed in Phosphate buffer saline (PBS) three times for 3 min and then allowed to react for 10 min with biotinylated anti‐mouse antibodies (LSAB kit, DAKO). Slides were rinsed with PBS and subsequently incubated with streptavidin‐HRP (horseradish peroxydase) (LSAB kit) for the next 25 min. After a 3‐min rinse in PBS, all slides were developed using the AEC substrate system and counterstained with 1% hematoxylin. Results for CD3^+^, CD4^+^, and CD8^+^ T were expressed as the percentage of positive cells relative to the total number of nuclei in the assessed area. For granzyme B and IFN‐γ, the percentage of positive cells in the dermis was expressed according to the semi quantitative grading system (the percentage of positive cells relative to the total number of nuclei in the area was recorded as: no staining; −, <10% of cells positive; +, 10‐50% of cells positive; ++.

Double staining for membrane molecules (CD4 or CD8) and intracellular molecules (GrB or IFN‐γ) was performed on five selected specimens using R.T.U VECTASTAIN UNIVERSAL ABS kit U (Vector laboratories, CA). First, anti‐Human GrB (clone GrB7, Dako) or anti‐Human IFNγ (Abcam, UK) was applied for 1h at room temperature followed by incubation with the R.T.U biotinylated PAN‐specific antibody (Vector laboratories) then streptavidin/peroxidase complex using AEC as a substrate (red). Subsequently, sections were blocked with normal horse serum for 20 min. Anti‐Human CD8 (clone C8/144B; Dako) or anti‐Human CD4 (clone 4B12; Dako) was applied as described above, using Vector SG as substrate (blue). Slides were counterstained with Methyl green. Slides were analyzed under a light microscope at a magnification ×400 by two independent investigators.

### 2.5 Statistical analysis

Data analysis was performed using Graph‐Pad Prism 5.03 for Windows. Nonparametric statistical test (Mann‐Whitney test) was used to compare levels of GrB, Grly and IFN‐γ mRNA between early and late ZCL lesions. Differences were considered statistically significant when *P* < 0.05. Correlation between continuous variables was evaluated with the Spearman's rank correlation test.

## 3 RESULTS

### 3.1 Clinical and histopathologic characterization of studied ZCL lesions

Skin specimens included in this study were from lesions with age ranging from 10 to 360 days and surface ranging from 78.5 to 1962.5 mm^2^ (mean ± SD, 614 ± 657.2 mm^2^). All the lesions, except one, were ulcerated and crusted. Histopathological analysis showed that most of them were characterized by a slight infiltration of the dermis with a moderate epithelial hyperplasia in conjunction with low frequencies of lymphocytes and polynuclear neutrophils (PNN) (± or +). Granuloma as well as giant and epithelioid cells were absent within the majority of lesion studied except for one lesion.

Quantification of KMP‐11 gene using RT‐qPCR showed the presence of *Leishmania* parasite within 12 lesion's specimens among the 16 studied (Table 1). The number of parasite ranged from 2 to 1162 parasites/10^5^ human cells.

### 3.2 Lymphocytic infiltrate within ZCL lesion

**Figure 1:**
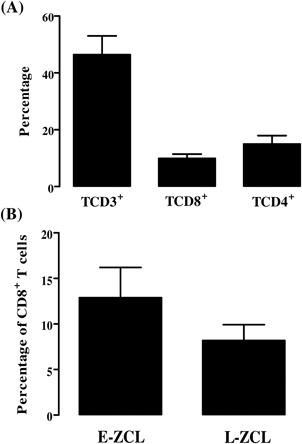
Frequency of CD3^+^, CD4^+^ and CD8^+^ T cells. (A) Histograms represent mean of percentage of CD3^+^, CD4^+^ and CD8^+^ T cells within the inflammatory infiltrate of ZCL skin lesions. Results are expressed as the percentage of positive cells relative to the total number of nuclei in the assessed area. (B) Percentage of CD8^+^T cells within early stage (E‐ZCL, duration ≤1 month) or late stage (L‐ZCL, duration >1 month) lesions

Analysis of the frequencies of CD3^+^, CD4^+^ and CD8^+^ T cells were performed by immunohistochemical study for fourteen ZCL lesion specimens. No NK cells (CD56^+^) were detected within these lesions. The percentage of CD3^+^ T cells among the lymphocytic infiltrate ranged from 25 to 97% while those of CD8^+^ T and CD4^+^T cells represent 5 to 19.3% and 7 to 27%, respectively (Figure 1A). Considering their age, the lesions were classified as early‐stage cutaneous leishmaniasis (E‐CL: one month of illness or less) or late‐stage cutaneous leishmaniasis (L‐CL: more than one month of illness). We found that the percentage of CD8^+^ T cells was slightly higher within E‐ZCL (mean ± SD,12.9 ± 6.6%) comparing to L‐ZCL (mean ± SD, 8.2 ± 4.8%) but the difference is not statically significant (Figure 1B).

### 3.3 Expression of cytotoxic granule's molecules and IFN‐γ within ZCL lesions

An eventual role of the cytotoxic cells within ZCL lesion establishment was explored by analyzing the intralesional expression of cytotoxic granule molecules (GrB and Grly) using RT‐qPCR. Results were referred, at the first time, to the expression of 18S ribosomal RNA (18S rRNA), as indicator of total cellular mRNA in each PCR reaction. All samples with C_T_ > 23 for 18S rRNA were excluded. Data were then expressed relatively to normal skin given by the formula 2^−ΔΔC^_T_ (ΔΔC_T_ is the difference between ΔC_T_ of the sample and the average of ΔC_T_ within normal skin).

**Figure 2:**
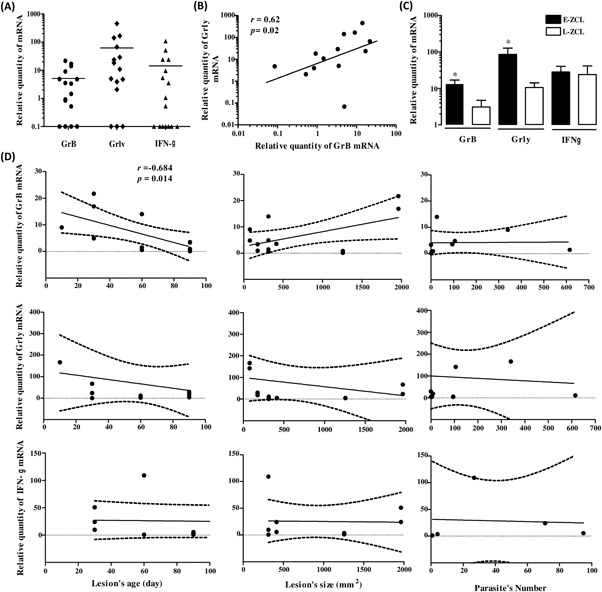
Expression of Granzyme B, granulysin and IFN‐γ within ZCL lesions. (A) Individual result of granzyme B, granulysin and IFN‐γ mRNA expression levels detected within lesion specimens. Results are expressed relatively to normal skin given by the formula 2^−ΔΔC^_T_ (ΔΔC_T_ is the difference between ΔC_T_ of the sample and the average of ΔC_T_ within normal skin). Horizontal bar indicates median (50th percentile) value of each group. (B) Correlation between GrB and Grly mRNA levels. (C) Comparison of GrB, Grly and IFNγ within E‐ZCL lesion (≤1 month of evolution, closed bar) or late L‐ZCL lesion (> month of evolution; open bar). Statistical comparisons were done using the Mann‐Whitney U‐test. *P < 0.05. (D) Relative levels of the different molecules (GrB, Grly, and IFN‐γ) represented as a function of different parameters related to the lesion (age, size of lesion and parasite's load). The graphs show the best fitted lines with 95% confidence interval. Statistical analyses were performed using Spearman's rank correlation test. Correlation coefficient “*r*” was considered significant when *P* < 0.05

Comparing to normal skin, GrB and Grly mRNA were detected at higher levels within 50% (8/16) and 80% (12/15) of ZCL lesions, respectively (Figure 2A). Interestingly, a positive correlation was found between levels of GrB and Grly (spearman's rank correlation coefficient *r* = 0.62; *P* = 0.02) (Figure 2B). To examine whether presence of these cytotoxic molecules was associated with pathogenesis of the ZCL lesion, we analyzed GrB and Grly levels considering age and surface of lesions. Accordingly, GrB levels were significantly higher in E‐ZCL compared to L‐ZCL (*P* = 0.016) (Figure 2C). Likewise, Grly levels were significantly higher in E‐ZCL comparing to L‐ZCL (*P* = 0.033) (Figure 2C). Moreover, a negative correlation was found between GrB levels and the age of lesion (spearman's rank correlation coefficient *r* = −0.685; *P* = 0.02) (Figure 2D). Contrariwise, no significant correlation was detected between levels of this molecule and lesion surface or parasite's number within the lesions.

Otherwise, IFN‐γ mRNA was detected within only 56% (9/16) of ZCL lesions tested (Figure 2A) and a positive correlation was found between IFN‐γ levels and those of GrB and Grly (spearman's rank correlation coefficient *r* = 0.905 and *r* = 0.738, *r*espectively). As shown in Figure 2D, no significant correlation was found between IFN‐γ and Grly mRNA levels with neither the age nor the size of lesion or parasite's load.

### 3.4 Detection of CD8^+^ T cells expressing GrB or IFN‐γ within lesion

**Figure 3:**
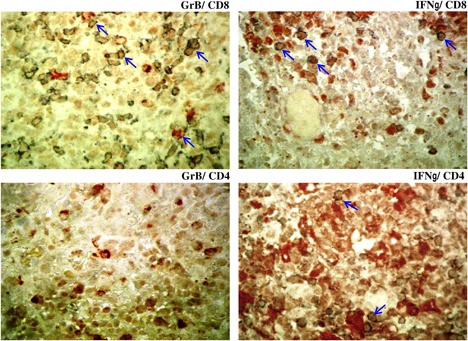
Granzyme B and IFN‐γ positive cells within ZCL lesions. Immunohistologic double staining using streptavidin‐peroxidase method with as a substrate, AEC (red) for GrB and IFN‐γ or Vector SG (bleue) for CD4 and CD8. Slides were counterstained with Methylgreen and analyzed at a magnification (×400). The arrows indicate the double‐stained cells

In attempt to confirm results obtained using the RT‐qPCR showing the expression of GrB and IFN‐γ within ZCL lesions we used the immunohistochemistry assay. Five specimens of ZCL skin lesions and three healthy skins were enrolled in this analysis. As shown in the Table 2 single staining showed the presence of GrB positive cells within the dermal infiltrate of ZCL lesions at a variable intensitiy. We noticed that GrB was also contained in intercellular spaces near lymphocytes in the epidermis. Likewise, a more intense staining was observed in the case of IFN‐γ. Contrariwise a mild staining was observed within the healthy skin specimens (data not shown). In attempt to characterize the phenotype of cells producing GrB and IFN‐γ, we performed a double‐staining immunohistochemical analysis specific for these molecules in addition to membrane marker (CD4 and CD8). We showed that most of cells positives for GrB were CD8^+^T cells whereas those positives for IFN‐γ were both CD4^+^ and CD8^+^T cells (Figure 3).

**Table 2:** Granzyme B and IFN‐γ expression within dermal infiltrate of ZCL lesions

	GrB^+^	IFN‐γ^+^
ZCL1	++	++
ZCL2	+	+
ZCL3	++	++
ZCL4	++	++
ZCL5	‐	+

−, no staining; +, <10% of cell positive; ++, 10‐50% of cells positive.

## 4 DISCUSSION

The relative ability of CD8^+^ T cells to contribute to protective or pathologic mechanisms in cutaneous leishmaniasis seems to be directly related to their effector functions. Accordingly, CD8^+^ T cells are protective when they produce IFN‐γ but promote pathology when they are cytolytic.^31^ In addition, there is strong evidence that CD8^+^ T cells are essential for the control of primary and secondary infection in visceral leishmaniasis.^15^ Nevertheless, the presence of these cells has also been associated with tissue damage in mucocutaneous leishmaniasis^20^ as well as during lesion progression in CL patients.^10^ Herein, we revealed the presence of CD8^+^T cells producing cytolytic molecules, GrB and Grly, but also IFN‐γ within ZCL lesions due to *L. major*.

The composition of inflammatory infiltrate in ZCL lesions was evaluated using conventional histological analysis. Lesions showed absence of either epitheloïd granuloma or giant and epitheloïd cells, indicating the ongoing process of intracellular parasite destruction. In fact, it is fully admitted that the development of granuloma is an indicator of the end of this process.^32^ The infiltrate is composed of moderate frequencies of polynuclear neutrophils (PNN), and same for plasmocytes indicating a minor implication of humoral immune response which is ineffective in intracellular parasitic infections.^33^ By contrast, a predominance of mononuclear cells within the infiltrate of lesions has been showed. Immunocytochemical study demonstrated variable percentages of CD3^+^, CD4^+^ and CD8^+^T cells with a slight predominance of CD4^+^ T cells over CD8^+^ T cells. The heterogeneity of CD4^+^ and CD8^+^ T cell frequencies have been already described in cutaneous lesions due to *L. braziliensis* 18 and *L. panamensis*.^34^ The predominance of CD4^+^ T cells that was observed within the majority of studied lesions could be related to the fact that they were sampled during the active phase of the disease. Indeed, a long‐term follow‐up of patients with CL due to *L. braziliensis* conducted by Da‐cruz and his collaborators had shown CD4/CD8 ratio superior to one during the active disease; however the healing process was associated with a decrease of CD4 and an increase of CD8, leading to comparable CD4 and CD8 proportions.^17^ Otherwise, a strong CD8^+^ T cell expansion has also been observed in *L. mexicana* patients during the healing process.^35^ Moreover, lesions of patients with localized cutaneous leishmaniasis (LCL) harbor a higher number of CD8^+^ T cells compared to patients with diffuse cutaneous leishmaniasis (DCL).^36^ While CD8^+^ T cells seem to correlate with cure in cutaneous and visceral forms of leishmaniasis, such cells seem to be implicated in the pathogenesis of mucocutaneous leishmaniasis (ML).^9^, ^16^, ^17^, ^18^ Our results showed that the percentage of CD8^+^ T cells was higher within early ZCL comparing to late ZCL lesion, but the difference between groups was not statistically significant. This might be confirmed by extending the number of E‐ZCL lesions.

There is evidence that CD8^+^ T cells may play an important role in the mechanisms for cure of or resistance to *Leishmania* infection, either through production of IFN‐γ and activation of macrophages or by a cytolytic effect of cytotoxic T lymphocytes (CTL) upon parasitized macrophages, or a combination of both effects.^17^ As advanced by Turner and collaborators, GrB is a serine protease emerging as an important mediator of skin injury, inflammation and repair. Found at low levels in healthy skin, GrB is dramatically elevated in chronic disease and inflammatory skin disorders, including diabetic ulcers, hypertrophic scarring, autoimmune skin disorders, cutaneous leishmaniasis and aging skin.^37^ Human cytotoxic granules contain also Grly, an antimicrobial peptide which selectively destroys cholesterol poor microbial membranes.^38^, ^39^, ^40^, ^41^ Together, Grly, perforine (PFN) and GrB are able to kill rapidly intracellular bacteria.^42^ It was recently demonstrated that GrB, delivered into infected host cells through the action of PFN and then into intracellular parasites by Grly, is able to kill *Trypanosoma cruzi*, *Toxoplasma gondii* and *Leishmania major*, in a PFN‐, Grly‐ and GrB‐dependent, but caspase‐independent, manner. GrB generate superoxide and inactivate oxidative defense enzymes to kill intracellular parasites.^43^ In our study, GrB and Grly mRNA levels were higher in ZCL compared to normal healthy skin indicating the presence of a GrB and Grly‐dependent cytotoxic activity and suggesting the outgoing of active process of parasite killing. However, no correlation was found between the mRNA expression of these molecules and, the parasite's load, nor lesion's size. Contrastingly, a negative correlation was found between GrB mRNA levels and lesion's age. Indeed, GrB mRNA expression levels were higher within E‐ZCL lesions compared to late stages (L‐ZCL) lesions. Such results are conflicting with those described for CL due to *L. braziliensis* showing that CD8^+^ T cells from late stage of CL (>10 days of evolution) expressed significantly higher levels of granzyme A than early stages of CL suggesting the involvement of such cells in lesion progression.^10^ This discrepancy could be explained by the fact that lesions enrolled in the latter study were classified in early stage CL with approximately 15 days of illness, non ulcerated lesions, or late‐stage LC with approximately 60 days of illness, ulcerated lesion. By contrast, the minimum age of lesions included in our study is one month except one lesion (10 days) and most of them were ulcerated. This discrepancy could also be attributed to the difference in the cytotoxic molecules targeted in both studies (Granzyme A vs granzyme B). Our choice of GrB was based on the results of Grosmann and his collaborators.^44^ These authors demonstrated that while half of circulating CD8^+^T lymphocytes and few circulating CD4^+^T lymphocytes coexpressed both granzymes A and B, activation of such cells induced substantial expression of granzyme B, but not granzyme A.^44^ Thus, the presence of cells producing GrB at higher levels within ZCL lesions may indicate the presence of activated CTL.

Besides their cytotoxic activity, CD8^+^ T cells are important source of cytokines.^25^, ^45^, ^46^ In human CL caused by *L. major*, CD8^+^ T cells and CD4^+^ Th1 cells are important for the resolution of the infection, mainly through the production of IFN‐γ.^18^, ^25^ IFN‐γ has been considered a critical cytokine involved in the pathogenesis of CL, due to its parasitical ability and also due to its inflammatory activity.^9^ These authors suggested that IFN‐γ is associated with disease progression which is in accordance with studies showing that the higher the frequencies of IFN‐γ or TNF‐α producing T lymphocytes, the larger the ulcerated lesion of CL patient.^47^ Other study revealed a dichotomy in human CL due to *L. braziliensis*: CD8^+^ granzyme B^+^ T cells mediate tissue injury, whereas CD4^+^ IFNγ^+^ T cells mediate parasite killing.^12^

Immunohistochemical analysis showed the presence of GrB positive cells within the dermal infiltrate of ZCL lesions. The phenotype of GrB‐producing cells was assessed using the double staining method. Since no NK cells were detected in ZCL lesions, we limited our study to the CD4^+^ and CD8^+^T cells and showed that GrB was produced essentially by CD8^+^T cells. However, such result needs to be confirmed by extending the sample's size of the studied lesions. Collectively our data showed the presence of cytotoxic cells within ZCL lesions due to *L.major* infection evidenced by the expression of GrB but also Grly mRNA at high levels compared to healthy skin. This indicates the involvement of such cells in the parasite's killing process inside the infected cells infiltrating the lesion and points to their implication in the parasite control.

## ETHICAL STATEMENT

The protocol was approved by the Bio‐Medical Ethics Committee (BMEC) of Pasteur Institute of Tunis by referring to the Declaration of Helsinki for institutional medical research including research on human material.

## CONFLICTS OF INTEREST

The authors declare no conflict of interest.
